# Diagnostic Value of Ultrasound-Guided Fine-Needle Aspiration for Pelvic Space-Occupying Lesions Via Rectal Endoscopic Ultrasound

**DOI:** 10.5152/tjg.2026.25487

**Published:** 2026-01-26

**Authors:** Xiaowei Fan, Chuyun Wang, Wei Wang, Shan Gao

**Affiliations:** 1 Department of Medicine, Wuhan University of Science and Technology, Hubei, China; 2 Department of Gastroenterology, Xiangyang Central Hospital, Affiliated Hospital of Hubei University of Arts and Science, Hubei, China; 3 Department of Cardiology, Nanzhang Hospital of Traditional Chinese Medicine, Hubei, China

**Keywords:** Biopsy, endoscopic ultrasound-guided fine-needle aspiration, pelvic tumor, EUS

## Abstract

**Background/Aims:**

The present study aimed to appraise the therapeutic outcomes of cystic lesion management and the diagnostic performance of endoscopic ultrasound-guided fine-needle aspiration (EUS-FNA) in characterizing pelvic space-occupying lesions.

**Materials and Methods:**

EUS-FNA was performed transrectally in 83 patients with pelvic space-occupying lesions identified by ultrasonography, computed tomography, or magnetic resonance imaging (MRI) between January 2016 and December 2024. Tissue was obtained for pathological analysis and smear cytology using a 19 G or 22 G puncture needle. Cytological samples were collected, and drainage was executed for pelvic cystic lesions. In the presence of an abscess, metronidazole solution was routinely provided for irrigation. Postoperatively, additional clinical monitoring was performed to assess assess patient prognosis, including the evaluation of clinical symptoms and laboratory indicators during follow-up.

**Results:**

After pathological and/or cytological evaluation of 83 patients, 67 instances of sil solid masses were identified, comprising 41 adenocarcinomas, 6 malignant mesenchymal tumors, 7 inflammatory masses, 3 lymphomas, 2 dermoid cysts, and 8 other cases. Sixteen instances of cystic masses were recorded, comprising 3 plasma cystadenomas, alongside 13 occurrences of perirectal abscesses, of which 11 patients with perirectal abscess were drained of pus and treated with metronidazole injection. The comparison of 19G and 22G puncture needles revealed no statistically significant variation in the percentage of punctures amenable to immunohistochemistry (*P* > .05). Thirteen patients with perirectal abscesses exhibited variable degrees of pain during EUS-FNA. Except for one patient who exhibited hematochezia attributable to a pelvic lesion in the prostate, none of the other patients encountered postoperative complications, including fever or hematochezia.

**Conclusion:**

The EUS-FNA is a direct, secure, and minimally invasive method with therapeutic promise for clarifying the characteristics of pelvic space-occupying lesions.

**Main Points**

- This retrospective study included 83 patients with pelvic masses who underwent transrectal EUS-FNA. It achieved high diagnostic efficacy for malignant tumors, with 94.4% sensitivity and 100.0% specificity.
- Transrectal EUS-FNA is safe safe, only 1 patient had hematochezia (successfully managed). No statistical difference in immunohistochemical eligibility existed between 19G and 22G needles (P > .05).
- Beyond diagnosis, EUS-FNA treated 11 perirectal abscesses via aspiration and metronidazole irrigation, achieving complete healing without recurrence during a 2-month follow-up.

## Introduction

Clinical observations indicate that pelvic masses may vary from benign entities, such as cysts or abscesses to malignant tumors, with their etiology being intricate. Due to its superior soft tissue contrast and resolution, computed tomography (CT) is the most commonly employed diagnostic technology in clinical settings. Notable advantages encompass the capacity to thoroughly delineate the extent of the occupied lesion and its relationship with adjacent tissues and organs, in addition to the capability of obtaining multidirectional images without necessitating patient repositioning. Describing the lesion is more arduous, despite the accessibility of imaging tests such as CT scans. Distinguishing between recurrent tumors and scar tissue is particularly problematic in patients who have received local surgery or radiation therapy. This complicates the accurate evaluation of the disease’s prognosis and, consequently, the formulation of a suitable treatment plan. The execution of accurate and effective treatment depends on obtaining a lesion sample for pathological diagnosis. Unnecessary surgeries resulting from misdiagnosis can also be averted.

Ultrasound-guided fine-needle aspiration (EUS-FNA) provides a more distinct ultrasound image of the lesion by meticulously assessing the gastrointestinal tract wall and surrounding organs. Cytological and histological specimens can be obtained using ultrasound-guided aspiration to secure a conclusive pathological diagnosis of the lesion. Nevertheless, the effectiveness of EUS-FNA in detecting pelvic mass lesions is still disputed owing to the intricacies of pelvic anatomy. Presently, there are only a few pertinent single-center studies and individual case reports. Transrectal EUS-FNA data from 83 cases were gathered and analyzed to examine the diagnostic and therapeutic utility of endoscopic ultrasonography for pelvic masses.

## Materials and Methods

### General Information

To determine the nature of the mass, a retrospective analysis of 83 cases of pelvic masses discovered by ultrasound, CT, magnetic resonance imaging (MRI), etc. at Xiangyang Central Hospital between January 2016 and May 2024 was conducted using transrectal EUS-FNA. Of these patients, 27 were male and 56 were female, with an age range of 29–86 years and a mean age of 58 years. The the maximum diameter of the lesions ranged from 1.9 to 11.8 cm, with a mean of 6.3 cm (Table 1). The primary sites of pelvic masses in 83 patients mainly included the rectum, uterus, ovaries, prostate, colon, and peritoneum (Table 2). Ethical committee approval was received from the Ethics Committee of Xiangyang Central Hospital (Approval number: 22024-062, Date: May 16, 2024). Informed consent was obtained.

### Inclusion and Exclusion Criteria

Patients aged 18 years or older who consented to or requested an EUS-FNA evaluation and provided written informed consent were eligible for study participation. Documentation of a pelvic mass on routine imaging constituted an additional inclusion criteria. Patients with serious cardiac or pulmonary conditions, those who couldn’t handle EUS-FNA/B, those who had a clear risk of bleeding, and those with mental illnesses who couldn’t comply with EUS-FNA were all excluded.

### Statistical Analysis

The data were analyzed using SPSS 26.0 (IBM SPSS Corp.; Armonk, NY, USA) statistical software. Fisher’s exact probability method was used to assess the count data, which were reported as percentages. A difference was considered statistically significant if it was less than 0.05.

### Operational Equipment and Puncture Needle Selection

The Pentax 3500 endoscopy mainframe, the Hitachi 5500 ultrasound mainframe, and the Japan Pentax EG-3630UT (probe frequency 5-10 MHz, electronic line array fan scanning) are used for ultrasound endoscopy. Selection of 19G puncture needles includes COOK Echotip-19 and 22G puncture needles: COOK Echotip-1-2 and Olympus NA-10J-1. The 22G puncture needle was chosen from Olympus NA-10J-1 and COOK Echotip-1-2, while the 19G puncture needle was chosen from COOK Echotip-19.

### EUS-FNA Procedure

The bowel was regularly cleaned prior to the procedure, and the patient or designated representative completed the appropriate informed consent form. In order to assess the size, shape, location, and echo intensity of the pelvic space-occupying lesion, the patient was in 1 of 3 positions during the examination: supine position, left lateral recumbent, or right lateral recumbent. The probe was positioned in the rectum to scan the pelvis, with the entry lens positioned 2-20 cm from the anus. The patient was then instructed to choose the best puncture position based on the endoscopic ultrasound image. The color Doppler function was used to examine the blood vessels along the puncture path, with particular attention paid to avoiding the large blood vessels. In order to match the puncture needle with the center of the mass, the clamp was raised during the puncture to change the direction. After confirming that the needle tip had penetrated the target lesion, the stylet was withdrawn and a negative-pressure syringe was attached.

Cystic solid masses were punctured and aspirated with a 22G puncture needle, puncturing and extracting the cystic fluid 1-2 times and puncturing the solid part of the body 2-3 times, with negative pressure of 0-10 mL, and lifting and inserting back and forth in the lesion 10-30 times. Abscesses or cystic fluid masses were punctured and aspirated 1-3 times with a 19G puncture needle, maintaining negative pressure of 5-10 mL. For the first time, abscesses or cystic fluid masses were punctured and aspirated 1–3 times with a 19G puncture needle, maintaining negative pressure of 5–10 mL, with a negative pressure of 0–10 mL applied and repeated to-and-fro manipulation performed within the lesion for 10–30 passes. If the initial aspiration yields a large volume of hemorrhagic fluid with minimal linear tissue, repeated negative-pressure manipulation was omitted. Once an adequate specimen is obtained, negative pressure is released and the needle is withdrawn.

Smear cytology, carcinoembryonic antigen, and bacterial culture were performed on puncture aspirates as needed. Using a needle, the extracts were pushed out onto a slide or into a liquid-based cytology preservation solution. If tissue strips developed, they were then fixed in a 10% formalin solution. Smear cytology, carcinoembryonic antigen, and bacterial culture were performed on puncture aspirates as needed. Repeat aspiration was performed 2–3 times if necessary. Postoperative follow-up was conducted for the pathological and cytological results.

### Postoperative Treatment

Patients were monitored for fever, blood in the stool, stomach pain, and other side effects following EUS-FNA. Inpatients received intravenous metronidazole or tinidazole for 2 days postoperatively as prophylactic anti-infective therapy; outpatients were administered oral norfloxacin capsules for the same duration. Inpatients were monitored daily for any adverse events for 3 dayas following surgery. Patients who were outpatients were monitored in the ultrasonography endoscopy room when they visited the hospital to acquire the results of their pathology tests. They visited the hospital at any time if there were any adverse events, such as fever and stomach trouble. For patients with an inconclusive diagnosis who required subsequent surgical intervention, surgical pathological examination was performed after the puncture procedure, with clinical follow-up extended for more than 2 months.

## Results

**Figure 1.**
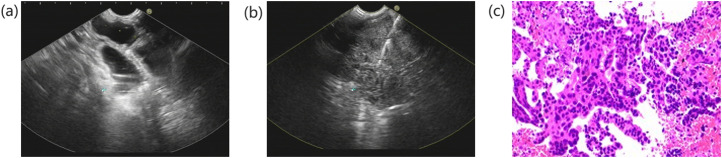
(a) A pelvic hypoechoic mass, approximately 9 cm in diameter, was discovered by ultrasound endoscopy. (b) A fine-needle aspiration biopsy was conducted on the pelvic mass under the guidance of endoscopic ultrasonography. (c) The solid pelvic mass’s pathologic results revealed cancer.

**Figure 2.**
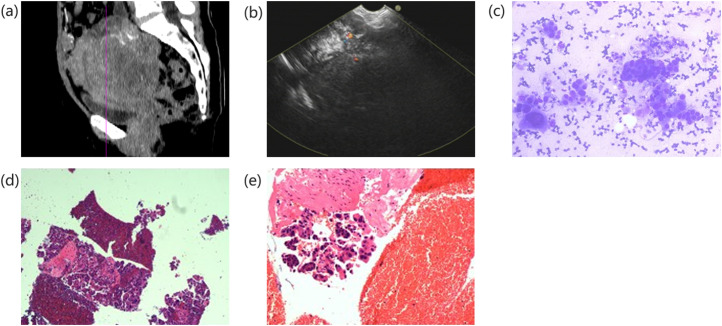
Metastatic adenocarcinoma of the pelvis, (a) A large soft-tissue mass measuring about 8.5 × 8.1 cm is seen in one pelvis on a sagittal CT scan. It has irregular morphology, unclear borders, and multiple patches of calcified foci inside. It also has heterogeneous density of enhancement within the uterus, inhomogeneous enhancement on enhancement, and unclear demarcation from the anterior-posterior uterine border. (b) According to EUS, there is a large hypoechoic pelvic lesion with patchy hyperechoic shadows scattered throughout. Its morphology is irregular, its borders are unclear, and its anechoic signals are localized (colored dots symbolize flow signals from color Doppler; red dots indicate blood flow toward us; blue dots represent blood flow away from us). (c) Adenocarcinoma was identified by combining microscopic pictures with hematoxylin-eosin staining. (d) (e) Pelvic adenocarcinoma accompanied by immunohistochemistry and morphology typical of high-grade plasma carcinoma. Vimentin (individually +), CA-125 (+), P16 (partially +), PR (spot foci weak +), ER (foci +), HNF-1 (-), Napsin-A (-), Villin (-), Ki-67 (Li: ~70%), CK7 (+), CK20 (-), CK18 (+), Pax-8 (+), WT-1 (+), and P53 (diffuse strong +, suggestive of mutant phenotype) were all detected by immunohistochemistry.

Tissue strips were taken from 79 cases that were sent for pathology and cytology, with the exception of 13 cases of perirectal abscess and 1 case of dermatoid cyst, which were only subjected to smear cytology. Eighty-three patients who were screened based on the nadir criteria underwent fine-needle aspiration biopsy. According to the pathological examination results, there were 67 cases of solid masses in the pelvis, 42 of which were adenocarcinomas (Figures 1 and 2), 6 of which were malignant mesenchymal stromal tumors, 7 were inflammatory masses, 3 were lymphomas, 1 was a dermoid cyst, and 8 were identified as other indeterminate cases. Of these, 8 cases were combined with laparoscopic or surgical exploration, and 3 of them turned out to be adenocarcinomas. Cytological examination of 67 patients with pelvic solid space-occupying lesions yielded a diagnostic sensitivity of 94.4%, specificity of 100.0%, positive predictive value of 100.0%, and negative predictive value of 81.2% for identifying pelvic malignancy. Transrectal EUS-FNA results of cases with substantial pelvic mass are shown in Table 3. Thirteen cases of perirectal abscesses and 16 cases of cystic or cystic-solid lesions, including 3 plasma cystic adenomas, were reported. Two of the cases had only a small amount of pus removed because the pus was so viscous, while the other 11 cases had metronidazole injections and repeated irrigations. The 11 cases were reexamined 2 months later, and the abscesses had vanished.

A 19G puncture needle was used in eight cases, and all punctures were sufficient to do immunohistochemical staining (100%). Of the 67 solid lesions, 59 were sampled with a 22G needle, with a successful sampling rate of 96.6% (57/59). There was no statistically significant difference in the percentage of punctures suitable for immunohistochemistry immunohistochemistry between the 19G and 22G puncture needles (*P *> .05) (Table 4).

Thirteen patients with perirectal abscesses experienced noticeable pain during the puncture procedure. The discomfort was quickly reduced following the puncture process, and none of them received any extra care. With the exception of one patient with metastatic adenocarcinoma whose pelvic lesion was in the prostate gland and who had blood in the stool, 83 cases were observed following the puncture procedure. The remaining patients did not exhibit any overt symptoms of abdominal pain, fever, hematochezia, or other adverse reactions.

## Discussion

The urinary bladder, urethra, rectum, and reproductive organs are the main organs of the pelvis. Lesions that occupy these organs are common clinical conditions caused by a variety of factors, such as abnormal body fluid accumulation, abnormal proliferation of pelvic tissues, and pelvic adhesions.^1^ The two types of pelvic lesions are benign and malignant. Although benign lesions frequently do not exhibit certain clinical symptoms and characteristics, they can cause internal swelling in the patient’s pelvis. In certain cases, these lesions are accompanied by several symptoms, which can significantly impact the patient’s life and work.^2^ Patients’ lives may be jeopardized if pelvic mass lesions are not treated promptly promptly, as it increases the chance of malignancy. The prognosis of individuals with pelvic masses can be effectively improved by early diagnosis.^3^

While EUS-FNA allows transrectal puncture biopsy of pelvic masses and the acquisition of tissue samples for pathological and cytological analysis, conventional imaging modalities including ultrasound, CT, and MRI fail to provide a conclusive diagnosis.^4^ Originally used primarily to diagnose pancreatic tumors, EUS-FNA has recently been used more frequently to diagnose masses outside the pancreas,^5^ including perigastric intestinal wall bulge,^6^ mediastinum,^7^ retroperitoneum,^8^ pelvic masses,^9^ and others. It possesses the ability to precisely diagnose a patient prior to surgery, pinpoint the nature of the tumor, differentiate primary from metastatic cancers, and detect potentially beneficial genetic changes to tailor the appropriate treatment plan.

In addition to being expensive, using CT-guided percutaneous puncture biopsy to determine the type of pelvic lesions is both challenging and time-consuming. Lesions that are larger in scope and closer to the abdominal wall are more suitable for the traditional ultrasound-guided percutaneous puncture biopsy.^4^ Transabdominal wall ultrasound is frequently inadequate for masses that are inaccessible through the abdominal wall and are often covered by intestinal loops which increases the risk of penetrating the bowel loops. Additionally, transabdominal ultrasound-guided percutaneous biopsies are not appropriate for patients with smaller pelvic masses that are challenging to accurately puncture through the abdominal wall. In addition to guiding the puncture needle to pierce the mass at the closest distance and providing real-time monitoring of the puncture process, a linear-array endoscopic ultrasound can clearly visualize pelvic lesions. At the same time, color Doppler imaging can identify vital adjacent organs and major blood vessels along the puncture path to reduce the risk of bleeding and other complications.^10^ In addition to diagnosing pelvic diseases, EUS-FNA/B can perform therapeutic procedures such as lavage and the puncture and drainage of perirectal abscesses, having a therapeutic function.

The process of acquiring tissue under the guidance of transendoscopic ultrasonography is safe. According to reports, its total complication rate ranges from 0.3% to 2.2%.^11^ Tumor cell implantation is the most concerning consequence. Nonetheless, there are currently few instances of EUS-FNA needle tract implantation.^12^ Clinicians may initiate symptomatic management based on the patient’s clinical status if the most common complications—hemorrhage, infection, and perforation—occur.^13^ Prophylactic antibiotics should be considered for transendoscopic ultrasound-guided fine-needle aspiration biopsies of cystic lesions to prevent infection. According to the American Society for Gastrointestinal Endoscopy (ASGE) guidelines, prophylactic antibiotics are not advised while doing EUS-FNA on solid lesions because there is a low risk of bacteremia associated with this procedure.^14^ Because of the presence of microorganisms in the intestinal lumen, the performance of EUS-FNA may still be complicated by a local infection, even with a small penetration needle. As a result, postoperative antibiotic prophylaxis for infection should be administered for 2 days, and preoperative bowel cleansing should be done regularly. No fever or infectious complications were identified in any of the 83 patients during postoperative follow-up. In this study, to prevent infection, inpatients received intravenous metronidazole or tinidazole for 2 days postprocedure, while outpatients were prescribed oral norfloxacin capsules for the same 2-day postoperative course; none of these 83 patients developed fever or infection during a 2-month postoperative follow-up period.

Changes in the cystic fluid’s echogenicity indicate the existence of intracapsular bleeding, which seldom progresses to a serious degree. Although bleeding normally stops on its own, several drugs that interfere with coagulation can make things worse. Coagulation parameters should be regularly evaluated before EUS-FNA to prevent bleeding. Clopidogrel should be stopped 7 days before the procedure, low-molecular-weight heparin should be held for 12–24 hours and unfractionated heparin held for 6 hours prior to the procedure. Aspirin does not need to be stopped. To prevent thrombotic events, high-risk patients should stop taking warfarin 5 days before surgery and start heparin bridging therapy.^15^ Of the 83 patients who underwent EUS-FNA in this study, only 1 patient—diagnosed with metastatic adenocarcinoma with a pelvic lesion located in the prostate—developed hematuria. The patient was transferred to the urology department for specialized management, after which the hematuria was promptly controlled. During the 2-month postoperative follow-up, the patient had no complications such as fever, infection, or bleeding.

Only a small number of studies have reported the use of EUS_FNA for pelvic lesions using the lower gastrointestinal tract approach, where the target lesion was limited to the rectum or perirectal area.^16^ The EUS-FNA is widely used as a standard technique for obtaining pathological specimens through the upper gastrointestinal tract and thus diagnosing peripheral lesions. In 2021, a single-center retrospective study conducted at Gifu University Hospital in Japan used database analysis to identify 49 patients who had undergone EUS-FNA/B for pelvic lesions between January 2008 and December 2018. Of these, 28 underwent the procedure via the upper gastrointestinal tract approach, and 21 underwent via the lower gastrointestinal tract approach. We treated pelvic lesions below the level of the internal and external iliac bifurcations with the lower GI approach, while we treated those at the level of the major iliac and internal and external iliac bifurcations with the upper GI method. With an overall success rate of 95.2% (20/21) for the lower gastrointestinal tract approach and 91.8% (45/49) to 89.3% (25/28) for the upper gastrointestinal tract method, the study had a comparatively high success rate.^17^ While there were no adverse effects in the trial, the ultrasound endoscope had to be pushed downward toward the duodenum throughout the procedure, and it is still necessary to further evaluate the safety of the upper gastrointestinal tract approach to pelvic lesions using EUS-FNA. To view pelvic lesions during the surgery, the endoscope must be moved downward toward the duodenum or gastric wall, significantly raising the risk of bleeding or perforation.

From September 2014 to December 2021, 35 patients with pelvic masses who were hospitalized at Soochow University’s Second Affiliated Hospital for EUS-FNA via the lower gastrointestinal tract route had their clinical data gathered for a 2022 study that was published in Translational Cancer Research. The pathological features of the surgical specimen were used to make a definitive diagnosis for 10 individuals who had surgery. The final diagnosis for the 25 patients who did not have surgery was determined by clinical follow-up or the malignant pathological findings of EUS-FNA. In identifying malignant pelvic masses, EUS-FNA’s sensitivity, specificity, positive predictive value, negative predictive value, and accuracy were, 91.3% (21/23), 100.0% (12/23), 100.0% (12/23), 100.0% (12/23), 100.0% (12/23), 100.0% (12/23), 100.0% (12/23), and 100.0% (12/23), respectively. The corresponding sensitivity, specificity, accuracy, positive predictive value, and negative predictive value were 91.3% (21/23), 100.0% (12/12), 100.0% (21/21), 85.7% (12/14), and 94.3% (33/35). All individuals experienced no complications from the biopsy puncture.^4^

Ultrasound endoscopy is becoming more and more useful for treating conditions like choledocholithiasis,^18^ perirectal abscess drainage,^19^ tissue glue injection of varices in the stomach fundus,^20^ EUS-guided blockade of the abdominal plexus for intractable abdominal pain in inoperable patients with advanced pancreatic cancer,^21^ and more. Thirty-seven patients had EUS-guided drainage of perirectal and sigmoid abscesses (31 postoperatively and 6 because of diseases like CD) using plastic or luminal-adjacent metallic stents in a 2-center case series research.^22^ On follow-up CT at 4 weeks, all patients had complete regression of the abscesses and a marked improvement in their symptoms. At 3 and 12 months, 3 patients experienced recurrences that necessitated surgical drainage. In the current study. In the current study, 11 patients with perirectal abscess underwent pus aspiration via puncture, followed by metronidazole injection for irrigation. During the 2-month follow-up, the abscess cavities were completely healed, and no complications such as fever, infection, or bleeding occurred. In addition to alleviating patient discomfort, it is clear that ultrasonic endoscopic drainage of pelvic abscesses is safe, has a good long-term prognosis, and can be taken into consideration as an alternative to percutaneous and surgical drainage.

The qualitative diagnosis of pelvic space-occupying lesions can be made with high accuracy with EUS-FNA, and the risk of associated consequences is quite low. It is a qualitative diagnostic method that has demonstrated safety, effectiveness, and feasibility for lesions that occupy pelvic space. It has a high clinical value and produces positive outcomes when used to treat pelvic abscesses. However, more proof is still required from multicenter studies with larger sample sizes.

## Figures and Tables

**Table 1.** Baseline Characteristics of the Patients

**Baseline Characteristics**	**Digital**
Number of patients, n	83
Age, years, average (range), n	58 (29-86)
Gender, male/female, n	27/56
Size of lesions, cm average (range)	6.3 (1.9-11.8)

**Table 2.** Primary Organs of Pelvic Masses Among 83 Patients

**Primary Organs**	**N**
Rectum, n	11
Uterus, n	6
Ovary, n	41
Fallopian tube, n	2
Prostate, n	14
Pelvic unorgan, n	9

**Table 3.** Transrectal EUS-FNA Results of Cases with Substantial Pelvic Mass (n = 67)

EUS-FNA Diagnosis	Type of Disease (Final Diagnosis) n = 67	
	Malignant Tumor	Non-Malignant Tumor
Malignant tumor	51	0
Non-malignant tumor	3	13

**Table 4.** Immunohistochemical Results Available for Pelvic Parenchymal Masses (n = 67)

Puncture Needle Type	Type of Disease (Final Diagnosis) n = 67	
	Immunohistochemistry Available	No Immunohistochemistry
22G	57	2
19G	8	0

## Data Availability

The data that support the findings of this study are available on request from the corresponding author.

## References

[1] Wu M. Application value of abdominal ultrasound and vaginal ultrasound in the diagnosis of pelvic masses. Chin J Mod Drug Appl. 2023;17(16):91 93.

[2] Gao K. Investigation of the role of abdominal ultrasonography in the clinical diagnosis of pelvic masses. J Imaging Res Med Appl. 2022;6(8):164 166.

[3] Shaw R Lokshin AE Miller MC Messerlian-Lambert G Moore RG. Stacking machine learning algorithms for biomarker-based preoperative diagnosis of a pelvic Mass. Cancers (Basel). 2022;14(5):1291. (doi: 10.10.3390/cancers14051291) PMC8909341 35267599

[4] Cai W Cheng G Tao F Hu D Wu W. Transrectal endoscopic ultrasound-guided fine-needle aspiration biopsy for qualitative diagnosis of pelvic space-occupying lesions: a diagnostic test. Transl Cancer Res. 2022;11(9):3267 3276. (doi: 10.10.21037/tcr-22-2057) 36237230 PMC9552266

[5] Kumar P Rana SS Kundu R Endoscopic ultrasound-guided fine-needle aspiration cytology in diagnosing intra-abdominal lesions. CytoJournal. 2022;19:56. (doi: 10.10.25259/Cytojournal_31_2021) PMC9699892 36447822

[6] Inoue T Okumura F Sano H Impact of endoscopic ultrasound-guided fine-needle biopsy on the diagnosis of subepithelial tumors: a propensity score-matching analysis. Dig Endosc. 2019;31(2):156 163. (doi: 10.10.1111/den.13269) 30171772

[7] Zhou J Cai T Wu D Chen X Wang F. The role of endoscopic ultrasound-guided fine-needle aspiration/biopsy in the diagnosis of mediastinal lesions. Front Surg. 2023;9:1065070. (doi: 10.10.3389/fsurg.2022.1065070) PMC9852620 36684177

[8] Pausawasdi N Maipang K Sriprayoon T Charatcharoenwitthaya P. Role of endoscopic ultrasound-guided fine-needle aspiration in the evaluation of abdominal lymphadenopathy of unknown etiology. Clin Endosc. 2022;55(2):279 286. (doi: 10.10.5946/ce.2021.218-IDEN) 34974679 PMC8995993

[9] Hayek K Kalinicheva T Shidham VB. Ultrasound-guided fine-needle aspiration of hyperenhancing lesion suspicious for pancreatic neuroendocrine tumor in the tail of pancreas-potential pitfalls. CytoJournal. 2017;14:8. (doi: 10.10.4103/1742-6413.205311) PMC5430498 28567109

[10] Hassan GM Paquin SC Albadine R Endoscopic ultrasound-guided FNA of pelvic lesions: A large single-center experience. Cancer Cytopathol. 2016;124(11):836 841. (doi: 10.10.1002/cncy.21756) 27448147

[11] Jenssen C Alvarez-Sánchez MV Napoléon B Faiss S. Diagnostic endoscopic ultrasonography: assessment of safety and prevention of complications. World J Gastroenterol. 2012;18(34):4659 4676. (doi: 10.10.3748/wjg.v18.i34.4659) 23002335 PMC3442204

[12] Kojima H Kitago M Iwasaki E Peritoneal dissemination of pancreatic cancer caused by endoscopic ultrasound-guided fine needle aspiration: a case report and literature review. World J Gastroenterol. 2021;27(3):294 304. (doi: 10.10.3748/wjg.v27.i3.294) 33519143 PMC7814364

[13] Mizuide M Ryozawa S Fujita A Complications of endoscopic ultrasound-guided fine needle aspiration: A narrative review. Diagnostics (Basel). 2020;10(11):964. (doi: 10.10.3390/diagnostics10110964) PMC7698484 33213103

[14] American Society for Gastrointestinal Endoscopy Standards of Practice Committee Khashab MA Chithadi KV Antibiotic prophylaxis for GI endoscopy. Gastrointest Endosc. 2015;81(1):81 89. (doi: 10.10.1016/j.gie.2014.08.008) 25442089

[15] Polkowski M Larghi A Weynand B Learning, techniques, and complications of endoscopic ultrasound (EUS)-guided sampling in gastroenterology: European Society of Gastrointestinal Endoscopy (ESGE) Technical Guideline. Endoscopy. 2012;44(2):190 206. (doi: 10.10.1055/s-0031-1291543) 22180307

[16] Han C Lin R Liu J Hou X Qian W Ding Z. Endoscopic ultrasonography-guided biopsy for differentiation of benign and malignant pelvic lesions: a systematic review and meta-analysis. Dig Dis Sci. 2015;60(12):3771 3781. (doi: 10.10.1007/s10620-015-3831-5) 26341351

[17] Mita N Iwashita T Senju A Endoscopic ultrasound-guided fine-needle aspiration of pelvic lesions via the upper and lower gastrointestinal tract approaches. BMC Gastroenterol. 2021;21(1):18. (doi: 10.10.1186/s12876-020-01582-8) PMC7788968 33407191

[18] Cominardi A Aragona G Cattaneo G Arzù G Capelli P Banchini F. Current trends of minimally invasive therapy for cholecystocholedocholithiasis. Front Med (Lausanne). 2023;10:1277410. (doi: 10.10.3389/fmed.2023.1277410) PMC10753828 38155666

[19] Khalid A Faisal MF. Endoscopic ultrasound-guided transrectal drainage of perirectal abscess in a patient with Crohn disease. Am J Case Rep. 2021;22:e930698. (doi: 10.10.12659/AJCR.930698) PMC8202418 34099613

[20] Giri S Patel RK Chavan R Endoscopic ultrasound-guided therapies versus retrograde transvenous obliteration for gastric varices: multicenter propensity matched analysis. Endosc Int Open. 2025;13:a25491165. (doi: 10.10.1055/a-2549-1165) PMC11996024 40230570

[21] Wilcox CM Bang JY Buxbaum J Effect of endoscopic ultrasound guided celiac plexus block on the palliation of pain in chronic pancreatitis (EPOCH Trial): study protocol for a randomized multicenter sham-controlled trial {1}. Trials. 2024;25(1):676. (doi: 10.10.1186/s13063-024-08478-y) PMC11472533 39396987

[22] Poincloux L Caillol F Allimant C Long-term outcome of endoscopic ultrasound-guided pelvic abscess drainage: a two-center series. Endoscopy. 2017;49(5):484 490. (doi: 10.10.1055/s-0042-122011) 28196390

